# Exploring Perceptions of Internet-Delivered Cognitive Behaviour Therapy among Public Safety Personnel: Informing Dissemination Efforts

**DOI:** 10.3390/ijerph17176026

**Published:** 2020-08-19

**Authors:** Hugh C. McCall, Angelo P. Sison, Jody L. Burnett, Janine D. Beahm, Heather D. Hadjistavropoulos

**Affiliations:** 1 Department of Psychology, University of Regina, 3737 Wascana Pkwy, Regina, SK S4S 0A2, Canada; Hugh.McCall@uregina.ca (H.C.M.); angelo.sison93@gmail.com (A.P.S.); Jody.Burnett@uregina.ca (J.L.B.); Janine.Beahm@uregina.ca (J.D.B.); 2 PSPNET, University of Regina, 2 Research Drive, Regina, SK S4T 2P7, Canada

**Keywords:** occupational injuries, internet, cognitive behavioural therapy, perception, telemedicine

## Abstract

*Background* Public safety personnel (PSP) experience high rates of mental health disorders but have limited access to treatment. To improve treatment access, there is a growing interest in offering internet-delivered cognitive behaviour therapy (ICBT) to PSP. As attitudes towards ICBT can both impact and inform ICBT implementation efforts, this study examines perceptions of ICBT among PSP who viewed a poster (a commonly used method of advertising ICBT) or a poster supplemented with a story of a PSP who benefitted from ICBT. *Methods* Participants (*N* = 132) from various PSP sectors were randomly assigned to view a poster or a poster and a story. Participants then completed an online survey assessing their perceptions of ICBT using both qualitative and quantitative questions. We used a mixed-methods approach to analyze the data. *Results* No differences in perceptions of ICBT were identified between the conditions. Ratings of credibility, treatment expectancy, anticipated treatment adherence, and acceptability suggested that PSP had positive perceptions of ICBT. Most participants (93%) reported that they would access ICBT if they needed help with mental health concerns. Participants ranked therapist-guided ICBT as their second most preferred treatment, with psychologists ranked first. Female participants found ICBT more credible than male participants. More experienced PSP reported lower acceptability and anticipated adherence to ICBT. *Conclusions* The findings suggest that many PSP are likely to be receptive to ICBT even when a simple poster is used as a method of informing PSP of this treatment option. Further attention to improving the perceptions of ICBT among certain groups may be warranted.

## 1. Introduction

Public safety personnel (PSP) include border security officers, correctional officers, dispatch/communication workers, paramedics, firefighters, and police officers. A study investigating the mental health of Canadian PSP found that 44.5% had screened positive for one or more mental health disorders, which contrasts with the diagnostic rate of 10.1% in the general population [1]. Past research on various PSP sectors has shown that PSP in other countries also struggle with high rates of mental health problems (e.g., [2,3,4]). PSP are routinely confronted with traumatic stressors as part of their regular duties [5] and have been shown to deal with high levels of work demand and physical and psychological stressors [5]. They regularly work overtime, face close public scrutiny, take part in labour and management conflicts, and routinely experience harassment [5]. PSP have experienced high rates of trauma exposure and mental health disorders but have avoided seeking psychological services, due, in part, to concerns about mental health stigma [6]. Despite successful initiatives to provide resiliency training and reduce the stigma surrounding mental health problems, such as the Road to Mental Readiness for First Responders program [7], a recent study found that many Canadian PSP still report experiencing limited workplace support for mental health-related problems [8]. PSP face several other barriers to mental health services, including long waiting times, distance from services, concerns about privacy and confidentiality, and cost of treatment [8,9].

Internet-delivered cognitive behaviour therapy (ICBT) is an alternative form of cognitive behaviour therapy (CBT) that is delivered online, often in the form of weekly lessons [10]. It can address common barriers to mental healthcare because it requires less therapist time than face-to-face treatments and can be accessed from virtually any location at any time [11]. Meta-analyses have found moderate to large effects of ICBT for several conditions, including major depression, generalized anxiety disorder, panic disorder, social anxiety disorder, and post-traumatic stress disorder [10]. Furthermore, there is mounting evidence that ICBT is effective when offered in routine practice [12,13], and studies have shown that ICBT with therapist support is comparably efficacious to face-to-face therapy [10]. ICBT may benefit both individuals and organizations by decreasing the amount of time people are off work due to mental health-related disability [14].

Despite its effectiveness, research suggests that ICBT is not perceived as positively as face-to-face therapy. Participants in prior studies have acknowledged the advantages of ICBT, such as convenience, flexibility, and anonymity [15,16], but have also perceived disadvantages such as low credibility and concerns about e-therapists’ ability to display empathy or build trust [16]. Participants in several studies have overwhelmingly expressed a preference for face-to-face therapy over web-based interventions [15,16,17]. Perceptions of ICBT as less helpful than face-to-face therapy could limit the utility of ICBT for expanding access to mental health treatment because treatment expectations are related to treatment outcomes in psychotherapy [18].

Negative perceptions of ICBT appear to result, at least in part, from a lack of awareness of and knowledge about ICBT. In one study [19], participants indicated that they would have a 48% likelihood of using computerized CBT if they were depressed and would expect it to lead to a 35% improvement in symptoms, on average. After watching an educational video, participants indicated that they would have a 70% likelihood of using computerized CBT and would expect a 60% improvement in symptoms. In addition to its effect on intentions to use treatments, mental health knowledge is related to lower levels of stigma among PSP [20]. However, past research has not examined how stories impact the perceptions of ICBT. Compared to other means of relaying information, stories allow for a more personal connection to and a deeper understanding of the information [21]. Prior research has shown that stories can have positive influences on advertising services and health communication [22,23].

The present study addresses four main research questions. First, how do PSP perceive ICBT after viewing educational materials? Second, do PSP who learn about ICBT from a poster and a story perceive ICBT more favourably than PSP who learn about ICBT from a poster alone? Third, do certain demographic or clinical characteristics predict perceptions of ICBT? Fourth, how would PSP prefer for ICBT to be delivered, especially in terms of therapist support and, particularly, with regard to therapist guidance? Regarding our first research question, based on the literature on educating people about ICBT, discussed above, we hypothesized that our sample would report relatively positive perceptions of ICBT after viewing the educational materials. Regarding our second research question, we hypothesized that the group presented with the poster and story would report more positive perceptions of ICBT than the group presented with the poster only. Our third and fourth research questions were exploratory; we expected that further examination of PSP’s perceptions of ICBT and the predictors of their perceptions could help inform education and implementation efforts (e.g., identifying specific client concerns about ICBT or groups who have elevated concerns).

## 2. Methods and Measures

### 2.1. Context

In 2019, the Government of Canada initiated a national Action Plan entitled Supporting Canada’s Public Safety Personnel: An Action Plan on Posttraumatic Stress Injuries [24]. Through this initiative, the Federal Government provided our research unit, PSPNET, with funding to develop and evaluate ICBT tailored for PSP. We began recruitment for the present study at approximately the same time as we launched our first ICBT program, called the PSP Wellbeing Course, in the province of Saskatchewan in January 2020. The PSP Wellbeing Course is based on a course initially developed at Macquarie University in Australia [25] and is designed to treat symptoms of depression, anxiety, and post-traumatic stress among PSP.

### 2.2. Participants

We recruited participants through email announcements sent to various PSP organizations across Saskatchewan, representing the following sectors: border security, corrections, communication/dispatch, emergency medical services, firefighting, and police. Invitations were also given in the form of unpaid advertisements on Facebook. We excluded 18 participants from data analysis for several reasons. Some participants (*n* = 7) indicated having previous experience with ICBT. One participant self-identified as a non-PSP, and another indicated that they had completed the survey twice. Lastly, some participants (*n* = 9) did not provide enough data to allow for meaningful data analyses. We analyzed data from 132 participants.

### 2.3. Procedure

Participants completed an online questionnaire, including demographic questions and questions about their recent mental health status. Participants were then randomly assigned to one of two groups. One group (*n* = 61) was presented with a poster describing the *PSP Wellbeing Course*, and the other (*n* = 71) was presented with the same poster along with a client story about a PSP using ICBT. The ICBT poster included a general description of ICBT being offered to PSP in Saskatchewan, including an overview of what ICBT is, the evidence for its effectiveness, and how the course is delivered. The poster is displayed in Appendix A. The ICBT client story followed a PSP character named Sam, whose story was derived from those of various PSP interviewed in a separate study [8]. Sam’s story described his experience using an ICBT program, including the knowledge, tools, and resources he received from ICBT and how they helped him manage his wellbeing and mental health as a PSP. The story is shown in Appendix B.

Both groups were then asked if they had any questions about ICBT based on the information. The group receiving the story was asked how much they related to the story and how much they expected other PSP would relate to the story. All participants completed a battery of questionnaires (described in detail below), including measures of the acceptability and credibility of ICBT and general help-seeking. We also administered questions pertaining to the participants’ perceptions of ICBT (e.g., preferences for the level of therapist support, general likes and dislikes). At the end of the survey, participants were given the option to click on a link to pspnet.ca if they wanted to learn more about ICBT for PSP.

### 2.4. Demographics Information

Participants answered questions regarding age, gender, education level, relationship status, the size of the community they live in, ethnicity, employment, years of experience in PSP roles, past-year psychotropic medication use, past-year mental health treatment, and knowledge and experience of ICBT.

### 2.5. Patient Health Questionnaire 4-Item (PHQ-4)

PHQ-4 [26] is a 4-item measure of depression and anxiety. Each item is answered on a 0–3 response scale, resulting in a total score ranging from 0 to 12. PHQ-4 has demonstrated good internal consistency and construct validity [26]. The Cronbach’s alpha for PHQ-4 was 0.87 in this study.

### 2.6. PTSD Checklist for DSM-5 (PCL-2)

PCL-2 [27] consists of two items related to symptoms of post-traumatic stress disorder (PTSD). Each item is answered on a 1–5 response scale. The measure has good specificity and sensitivity [27]. The Cronbach’s alpha for PCL-2 in this study was 0.88.

### 2.7. Story Relatability

Participants in the poster and story condition answered two questions about the relatability of the story: “Do you feel Sam’s experiences are similar at all to your own?” and “Do you feel Sam’s experiences are similar to any first responders/public safety personnel you know?”. Participants responded using a 0–100% response scale.

### 2.8. Treatment Acceptability and Adherence Scale (TAAS)

TAAS [28] is a measure assessing the acceptability of a treatment and a respondent’s anticipated adherence to that treatment. It is composed of ten questions, which respondents answer via a 7-point Likert scale. The items are summed with scores ranging from 10–70, with higher scores representing greater acceptability and anticipated adherence to the treatment in question [28]. The Cronbach’s alpha for TAAS was 0.86 in this study.

### 2.9. General Health Seeking Questionnaire (GHSQ)

GHSQ [29] was used in this study to assess the respondent’s perceived likelihood of seeking various sources of help. Response options ranged from 1 (extremely unlikely) to 7 (extremely likely). GHSQ has demonstrated good reliability and validity [29].

### 2.10. Credibility and Expectations Questionnaire (CEQ)

CEQ [30] is a questionnaire assessing the perceived credibility and expected outcomes of a treatment. It includes four items employing 9-point Likert scales and two items that respondents answer by selecting a percentage between zero and 100. We measured the credibility of treatment by calculating the mean of the first three items and expectancy by using the last item [31]. CEQ has demonstrated high internal consistency and good test–retest reliability [30]. The Cronbach’s alpha for the credibility subscale of CEQ was 0.86 in this study.

### 2.11. ICBT Treatment Support Preference Questionnaire

This bespoke questionnaire consists of four items regarding preferences related to therapist support. First, we asked, “Do you think ICBT is a mental health support you would access if you needed help?” and participants selected “yes” or “no”. We then asked participants how often they would like therapists to check in on their progress by email and how often they would like to send emails to e-therapists. Response options included “never”, “only if I [request/feel like] it”, “once a week”, and “twice a week”. Lastly, we asked participants to indicate whether they would prefer to complete ICBT with no therapist involvement, therapist involvement on demand, a therapist who checks the website and responds to questions once per week, or a therapist who does so twice per week.

### 2.12. Treatment Preference

We presented participants with 12 treatment options and asked them to rank the three options they would most prefer if they were dealing with depression, anxiety, or PTSD. The 12 options were as follows: ICBT with therapist support, ICBT with no therapist support, online counselling, psychologist, social worker, counsellor, doctor/GP, nurse practitioner, psychiatrist, self-help book, website information, and other (please specify). We also allowed participants to indicate if they “would not seek help from anyone”.

### 2.13. ICBT Likes and Dislikes

Using open-ended text boxes, we asked participants what they liked and disliked about ICBT, about their perceptions of the advantages and disadvantages of ICBT, and whether they had any questions about ICBT.

### 2.14. E-Therapy Assessment Measure (ETAM)

ETAM [32] consists of three items related to the perceived effectiveness of ICBT, appropriateness of ICBT, and preference for ICBT compared with conventional face-to-face therapy. Responses are rated on a 5-point Likert scale ranging from “Disagree Strongly” to “Agree Strongly”. We presented participants with an additional item using the same response scale: “In case of mental health problems, I would attend ICBT”.

### 2.15. Analysis

We employed a mixed-methods approach to data analysis. We conducted all quantitative analyses using SPSS (version 23). We used descriptive statistics to examine the background characteristics of the sample, overall perceptions of ICBT, and the relatability of the story for those assigned to the poster and story condition. We compared conditions on demographic and clinical characteristics using *t*-tests and chi-square analyses. In order to test our hypothesis that participants presented with the poster and story would report more positive perceptions of ICBT than participants presented with the poster alone, we conducted an ANOVA to examine whether the perceptions of ICBT (measured via CEQ, ETAM, and TAAS) differed between conditions. We also conducted three linear regressions to examine the predictors of the perceived credibility (measured via CEQ), treatment expectations (measured via CEQ), and acceptability and anticipated adherence (measured via TAAS) of ICBT. Predictors inputted into each regression analysis included age, gender, size of home community, PSP sector, years of experience as a PSP, education level, familiarity with ICBT, relationship status, medication, mental health treatment, and PHQ-4 and PCL-2 scores. We used descriptive statistics to examine how ICBT ranked in preference compared to other treatments and how PSP indicated they would prefer ICBT to be delivered.

We supplemented these quantitative analyses with qualitative analyses to further explore PSP’s perceptions of ICBT. Specifically, we used an inductive qualitative content analysis [33] to examine participants’ responses concerning their likes and dislikes of ICBT and their questions about ICBT. First, the author A.S. reviewed participants’ responses to the open-ended questions and developed a coding guide for participants’ perceived likes and dislikes of ICBT. Next, A.S. and authors H.M. and J.D.B. reviewed and refined the initial coding guide. All authors then met to finalize the coding guide, and H.M. and J.D.B. recoded several responses and reviewed all data to ensure it was coded consistently with the finalized coding guide.

### 2.16. Power Analysis

We conducted power analyses using G*Power 3 [34]. For our multiple regression analysis, given an alpha of 0.05, a power level of 0.80, and 12 predictors, and assuming a medium effect size of *f*^2^ = 0.15, we required a sample of 127 participants. For the ANOVA analysis, given an alpha of 0.05, a power level of 0.80, and two conditions, and assuming a medium effect size of *f* = 0.25, we required a sample size of 128.

## 3. Results

### 3.1. Participant Characteristics

Participants had an average age of 39.90 (*SD* = 9.54) years. The gender ratio was relatively evenly split between male and female PSP (male *n* = 68; 52%; female *n* = 62; 47%; nonbinary *n* = 2; 2%). Approximately half the participants (*n* = 70, 53%) reported living in communities with a population over 100,000 and half (*n* = 62, 47%) in communities under 100,000. Most PSP were married (*n* = 89, 67%), had attained a postsecondary degree (*n* = 85, 64%), and identified themselves as white (*n* = 119, 90%). Only 5 (4%) reported not being employed at the time of survey completion, and most (*n* = 86, 65%) had 10 or more years of experience working as PSP. The sample included PSP from various sectors: border security (*n* = 10, 8%), corrections (*n* = 18, 14%), communications/dispatch (*n* = 8, 6%), emergency medical services (*n* = 41, 31%), firefighting (*n* = 20, 15%), and police (*n* = 35, 27%).

About a quarter of participants (*n* = 32, 24%) reported using medication for a mental health problem within the previous year, and nearly half (*n* = 59, 45%) reported receiving professional help for a mental health problem within the previous year. When asked how familiar they were with ICBT, many participants (*n* = 56, 42%) indicated that they had at least “a little knowledge” of it. Participants had a mean PHQ-4 score of 3.4 (*SD* = 3.0), and most (*n* = 73, 55%) had clinically significant scores (i.e., scores greater than 3 [26]). The mean PCL-2 score was 4.25 (*SD* = 2.01), and most participants (*n* = 81, 61%) had clinically significant scores (i.e., scores greater than 4 [27]). Chi-square analyses and *t*-tests revealed no differences between conditions on any demographic or clinical characteristics (*p*s > 0.05). The demographic and clinical characteristics, collapsed across conditions, are summarized in Table 1.

### 3.2. Quantitative Analyses

To test our hypothesis that participants presented with the poster and story would report more positive perceptions of ICBT than participants presented with the poster alone, we used an ANOVA to compare the conditions on attitudes towards ICBT. The results indicated there were no differences on the expectancy subscale of CEQ (*F*(1, 130) = 0.198, *p* = 0.657), the credibility subscale of CEQ (*F*(1, 130) = 0.406, *p* = 0.525), ETAM (*F*(1, 130) = 0.001, *p* = 0.979), or TAAS (*F*(1, 130) = 0.006, *p* = 0.94). Given that demographic and clinical characteristics, credibility, expectancy, and e-therapy measure scores did not differ across conditions, both conditions were collapsed for the remainder of the analyses.

To test our hypothesis that participants would report relatively positive perceptions of ICBT, we calculated descriptive statistics to examine participants’ attitudes towards ICBT. Across both conditions, participants provided mean scores of 18.52 on the credibility subscale of CEQ (*SD* = 4.87), 53.79 on the expectancy subscale of CEQ (*SD* = 22.16), 9.20 on ETAM (*SD* = 3.19), and 51.86 on TAAS (*SD* = 9.42). On average, participants randomly assigned to review the story reported finding it to be 50% similar to their own experiences (*SD* = 30%) and 73% similar to the experiences of other PSP they knew (*SD* = 28%). When participants were asked to rank their top three most preferred sources of treatment, psychologists were most frequently ranked in the top three (*n* = 77, 58%), followed by therapist-guided ICBT (*n* = 68, 52%), counsellors (*n* = 50, 38%), and doctors (*n* = 47, 36%). See Table 2 for details. Results from the ICBT Therapist Support Preferences Questionnaire indicated that most participants (*n* = 123, 93%) would access ICBT if they needed help.

To explore our research question concerning possible predictors of perceptions of ICBT, we conducted three regression analyses. Clinical and demographic variables did not significantly predict scores on the expectancy subscale of CEQ (*R*^2^ = 0.10, *F*(13, 113) = 0.98, *p* = 0.48), but they did significantly predict scores on the credibility subscale of CEQ (*R*^2^ = 0.20, *F*(13, 113) = 0.2.19, *p* = 0.014). This second regression showed that identifying as female significantly predicted higher scores on the credibility subscale of CEQ (β = 0.29, *t*(113) = 3.26, *p* = 0.001). Lastly, a third regression showed that the clinical and demographic variables predicted TAAS total scores (*R*^2^ = 0.20, *F*(13, 113) = 2.20, *p* = 0.014). In this regression, years of experience as a PSP negatively predicted TAAS total scores (β = −0.23, *t*(113) = −2.22, *p* = 0.03). The results of these regressions are summarized in Table 3 below.

Results from the ICBT Therapist Support Preferences Questionnaire addressed our research question concerning PSP’s preferences for the delivery of ICBT. Many expressed a preference to have a therapist check in on their progress by email once (*n* = 84, 64%) or twice (*n* = 15, 11%) per week, although approximately one in four (*n* = 32, 24%) indicated that they would prefer therapists to be available upon request. Similarly, most participants indicated that they would prefer to have a therapist monitor their progress and respond to questions once (*n* = 55, 42%) or twice (*n* = 31, 23%) per week, and one in three indicated a preference for no monitoring and guidance on demand instead (*n* = 42, 32%). About half of our participants (*n* = 66, 50%) responded that they would email a provider “only if [they] feel like it”, followed by once per week (*n* = 52, 39%). Few (*n* = 9, 7%) indicated that they would email their therapists twice per week.

### 3.3. Qualitative Analyses

When invited to ask questions about ICBT, several participants (*n* = 7, 5%) asked logistical questions. For example, participants asked what would happen if users did not complete therapy, how many sessions users typically complete per week, and how long it takes to complete the program. Participants also asked questions about anonymity (*n* = 1, 1%) and costs (*n* = 1, 1%).

The most common “like” identified with ICBT was accessibility (*n* = 111, 84%). The theme of accessibility was subcategorized into seven categories, as presented in Table 4. The most frequently endorsed subcategories were convenience (*n* = 25, 19%) and the time-flexible nature of ICBT (*n* = 37, 28%). The two most common likes after accessibility were anonymity/privacy (*n* = 16, 12%) and that ICBT provides information/techniques/advice on mental health (e.g., “covers a large range of issues”; *n* = 14, 11%). The most common “dislike” was the lack of face-to-face interaction with a therapist or the belief that ICBT would feel impersonal (*n* = 41, 31%). Other participants expressed concerns about the effectiveness of ICBT (*n* = 11, 8%) or issues related to clients’ accountability and motivation (*n* = 14, 11%).

## 4. Discussion

### 4.1. Principal Findings and Implications

Overall, as hypothesized, participants reported positive perceptions of ICBT. Most participants (93%) indicated that if they needed help with mental health concerns, they would use ICBT. Across conditions, participants’ responses to CEQ and TAAS indicated that they perceived ICBT as moderately credible and acceptable and expected adequate adherence and moderate symptom improvement. Mean scores on CEQ were comparable to those of primary care patients and postsecondary students who were asked about their perceptions of ICBT in previous studies, and mean TAAS scores were slightly higher [35,36]. It is important to highlight that therapist-guided ICBT was the second most preferred treatment. It was included among the three most preferred treatments for 52% of participants, closely following psychologists, who were included among the three most preferred treatments by 58% of participants. This finding contrasts with recent studies in which participants have shown strong preferences for other treatments over ICBT [15,16,17]. The results of the ANOVA did not support our hypothesis that receiving additional information about ICBT through a story would result in more positive perceptions of ICBT compared to the group that only received an ICBT poster.

Participants provided useful feedback on how they would like ICBT to be delivered. Only 19% of participants ranked unguided ICBT as one of their three most preferred treatment options, and only 5% ranked it as their most preferred option, suggesting that therapist guidance is important to most PSP. Prior research has shown that the general public also prefers guided ICBT to unguided ICBT [37]. Most participants (65%) indicated a preference for therapists to check in on their progress at least once a week, although approximately one in three (32%) expressed a preference for optional therapist guidance over regular therapist check-ins.

We found two statistically significant predictors of perceptions of ICBT in our regression analyses. First, we found that a greater number of years of experience working as a PSP predicted lower acceptability of and anticipated adherence to ICBT on TAAS. Second, we found that female participants found ICBT more credible than male participants on CEQ. This second finding is consistent with previous literature showing that females are more likely to report positive perceptions of psychotherapy [38]. However, other studies that have examined participant attitudes toward ICBT did not find that gender predicted attitudes [35,39]. Likewise, previous studies have found that several other demographic and clinical variables have predicted attitudes towards ICBT. For example, one study [17] showed that older age, previous experience with Internet-based therapy, and confidence with computers, technology, and the internet predicted intentions to use internet-based therapy in the future. A recent study conducted in Saskatchewan found that higher perceived need for treatment, lower self-stigma regarding help-seeking, lower access to other forms of care, and lower computer anxiety predicted greater interest in ICBT [40]. Another recent study showed that among students, greater depression predicted lower acceptability and anticipated adherence on TAAS [36].

The most common perceived advantages of ICBT were convenience, the time-flexible nature of ICBT, the fact that no transportation is required, and anonymity/privacy. These findings are consistent with previous studies in which participants have recognized that ICBT can address common barriers to treatment, such as time, location, and anonymity [15]. The most common dislikes of ICBT were the lack of face-to-face interaction with a therapist and ICBT feeling impersonal. Participants also expressed concerns about the effectiveness of ICBT and the issue of client accountability and motivation. The participants’ concerns with ICBT were similar to those expressed by the general population in past research [15,17,41]. Few participants indicated that they had questions about ICBT. The questions participants asked were generally logistical in nature (e.g., about anonymity, costs, and timeframe). These questions may indicate important areas of curiosity or concern that ICBT researchers or providers should address when preparing educational information regarding ICBT for PSP.

Taken together, these findings have three important implications. First, they suggest that ICBT may be particularly well suited to PSP. The finding that therapist-guided ICBT was the second most preferred treatment was striking because prior research has shown that the general population strongly prefers other mental health treatments to ICBT [15,16,17]. The participants’ positive perceptions of ICBT suggest that ICBT may have good uptake among PSP. Furthermore, positive perceptions of treatments are related to better treatment outcomes [18]. Second, the present findings provide guidance on how researchers and providers of ICBT should educate PSP about ICBT. Specifically, the findings suggest that adding a client story to an educational poster does not improve PSP’s perceptions of ICBT. Additionally, few clients indicated that they had questions about ICBT after viewing the poster, suggesting that it provides a sufficient level of detail for most PSP. The questions participants asked were generally related to the logistics of ICBT, indicating that educational materials should clearly explain how ICBT is delivered. Male PSP and more experienced PSP may have less positive perceptions of ICBT; thus, it could be particularly important to design educational materials that appeal to PSP with these characteristics. Third, these findings provide guidance on how ICBT should be delivered to PSP. Participants demonstrated a greater preference for therapist-guided ICBT than self-guided ICBT. They indicated diverse preferences for therapist contact (e.g., once weekly, twice weekly, on demand), suggesting that providing clients with options may be more effective than giving all clients the same level of therapist contact. Participants also provided their perspectives on the disadvantages of ICBT (e.g., it could feel impersonal), which future research can help address.

Our research unit (PSPNET) recently conducted a study [8] in which we interviewed PSP about several topics, including their perspectives on ICBT. Despite some overlap in research questions and findings between the two studies, the recent study included only a qualitative analysis of the interviews with PSP, while the current study includes quantitative and qualitative analyses of survey data. Due in part to these methodological differences, the present study’s findings expand upon those of the recent study in six important ways. First, 93% of participants in the present study indicated that they would use ICBT if they needed help with a mental health problem, which contrasts with the finding in our recent study that only 62% of participants believed that PSP would use ICBT. Second, we did not ask participants to rank their most preferred treatments in the recent study, and the finding that ICBT was participants’ second most preferred treatment in the current study is novel. Third, the current study indicates that PSP have diverse preferences with respect to the regularity and frequency of therapist contact in ICBT. Fourth, we compared two means of informing PSP about ICBT in the current study, finding that adding a story did not result in more positive perceptions of ICBT. Fifth, we assessed for differences between PSP sectors in the current study, finding that there were no differences in perceptions among sectors. Sixth, we evaluated potential predictors of perceptions in the current study, finding that male and more experienced PSP may have less positive perceptions of ICBT.

### 4.2. Strengths, Limitations, and Future Research

This study has several strengths. First, it is unique, as it is one of the first to study PSP’s perceptions of ICBT. Second, our sample included many participants reporting clinically significant mental health concerns, who represent the population most likely to benefit from ICBT for PSP. Third, our sample was diverse in certain respects (e.g., community size, level of education, age, relationship status, PSP sector), which serves to increase the generalizability of our findings. Fourth, this study benefited from a mixed-methods approach, and we were able to address our research questions through both quantitative and qualitative lenses.

This study also has several limitations. It is possible that results were influenced by sampling bias (i.e., participants’ perceptions of ICBT may differ from those of PSP who decided not to participate). We cannot conclude that stories are not effective for educating PSP on ICBT; it is possible that a different story or an alternative method of presenting a story (e.g., video) would have had a greater impact on the participants’ perceptions, particularly in light of evidence that stories presented in an audio or video format are more persuasive than stories relayed through text [23]. Additionally, nearly half of our sample reported having at least “a little knowledge” of ICBT before viewing the educational materials, and participants’ preexisting perceptions of ICBT may have limited the impact of the educational materials. It is possible that additional factors we did not measure could have predicted participants’ perceptions (e.g., confidence in using technology). Finally, our sample was drawn exclusively from the province of Saskatchewan, and our findings may not generalize to other provinces or countries.

The results of this study provide several directions for future research. Future research on PSP perceptions of ICBT could benefit from sampling outside of Saskatchewan to assess whether perceptions differ in other provinces or countries. Future research could further explore strategies for overcoming disadvantages. Relatedly, in our study, male PSP perceived ICBT as less credible than female PSP, and PSP with more experience perceived ICBT as less acceptable and anticipated that they would have lower adherence. Thus, future research could explore possible solutions to improve the perceptions of ICBT among male and more experienced PSP. Additional research could also help identify and evaluate alternative strategies (e.g., videos) for educating PSP about ICBT. Future research will be required to examine the effectiveness of ICBT for PSP and the perceptions of ICBT among PSP who have used it.

## 5. Conclusions

PSP experience high rates of mental health disorders, and ICBT is well situated to help expand access to treatment for PSP. After viewing educational materials on ICBT, PSP who participated in this study reported positive perceptions of ICBT and a preference for ICBT over most other sources of mental healthcare. Female participants perceived ICBT to be more credible than male participants did. More experienced PSP reported lower acceptability and anticipated adherence. There was no difference in perceptions between participants who received ICBT information from a poster and those who received ICBT information from a poster and a story. Participants in both conditions reported positive perceptions of ICBT, which supports the use of posters as a simple method of informing PSP about ICBT. Developers and providers of ICBT for PSP should be aware that our findings indicate that PSP have diverse preferences concerning the delivery of ICBT. Future research can extend these findings in several ways, such as by exploring strategies to improve perceptions of ICBT among groups with less favourable perceptions.

## Figures and Tables

**Table 1:** Participant demographic and clinical characteristics.

Participant Characteristics	All Participants (*N* = 132)
Continuous variables, *M* (*SD*)	
Age	39.90 (9.54)
PHQ-4 total score	3.49 (3.05)
PCL-2 total score	4.26 (2.02)
Categorical variables, *n* (%)	
Gender	
Male	68 (52)
Female	62 (47)
Nonbinary	2 (2)
Community size	
<100,000	62 (47)
>100,000	70 (53)
Relationship status	
Not married/partnered	43 (32)
Married/partnered	89 (67)
Ethnicity	
Ethnic minority	13 (10)
White	119 (90)
Employment status	
Not working	5 (4)
Working	126 (94)
Years of PSP experience	
0–10 years	45 (34)
10+ years	86 (65)
Highest level of education	
No degree	47 (36)
Degree	85 (65)
PSP sector	
Border security	10 (8)
Corrections	18 (14)
Dispatch/communications	8 (6)
Fire	20 (15)
Paramedicine	41 (31)
Police	35 (27)
Medication for mental health in last 12 months	
Yes	32 (24)
No	100 (76)
Professional help for mental health in last 12 months	
Yes	59 (45)
No	73 (55)
Familiarity with ICBT	
Not familiar	76 (58)
Familiar	56 (42)
PHQ-4	
Not clinically significant (less than 3)	59 (45)
Clinically significant (3 or greater)	73 (55)
PCL-2	
Not clinically significant (less than 4)	51 (39)
Clinically significant (4 or greater)	81 (61)

Note. PHQ-4 = Patient Health Questionnaire 4-Item; PCL-2 = PTSD Checklist for DSM-5; PSP = public safety personnel; ICBT = internet-delivered cognitive behaviour therapy.

**Table 2:** Mental health treatment preferences among public safety personnel.

Treatment Preference		Most Preferred Treatment, *n* (%)	Second Most Preferred Treatment, *n* (%)	Third Most Preferred Treatment, *n* (%)	One of Three Most Preferred Treatments, *n* (%)
1.	Psychologist	45 (34)	22 (17)	10 (8)	77 (58)
2.	ICBT with therapist assistance	20 (15)	19 (14)	29 (22)	68 (52)
3.	Counsellor	17 (13)	15 (11)	18 (14)	50 (38)
4.	Doctor	12 (9)	15 (11)	20 (15)	47 (36)
5.	Psychiatrist	7 (5)	15 (11)	8 (6)	30 (23)
6.	ICBT with no therapist assistance	7 (5)	11 (8)	7 (5)	25 (19)
7.	Online counselling	2 (2)	9 (7)	10 (8)	21 (16)
8.	Self-help book	2 (2)	5 (4)	9 (7)	16 (12)
9.	Website Information	3 (2)	5 (4)	5 (4)	13 (10)
10.	Other (e.g., priest, significant other)	6 (5)	2 (2)	1 (1)	9 (7)
11.	Social Worker	1 (1)	4 (3)	2 (2)	7 (5)
12.	Nurse Practitioner	1 (1)	2 (2)	3 (2)	6 (5)

**Table 3:** Summary of regression models and statistically significant predictors.

Model and Significant Predictors	*B*	95% CI for *B*		*SE B*	β	*p*	*R* ^2^	Δ *R*^2^
		Lower Limit	Upper Limit					
Predicting CEQ (expectancy)						0.48	0.10	0.00
Constant	75.04	33.33	116.76	21.06		0.001		
Predicting CEQ (credibility)						0.014	0.20	0.11
Constant	17.64	9.18	26.10	4.27		0.001		
Identification as female	2.72	1.07	4.37	0.83	0.29	<0.001		
Predicting TAAS						0.014	0.20	0.11
Constant	59.71	43.48	75.95	8.19		<0.001		
Years of experience as PSP	−4.29	−8.13	−0.46	1.94	−0.23	0.03		

Note: CI = confidence interval; *SE* = standard error; CEQ = Credibility and Expectations Questionnaire; TAAS = Treatment Acceptability and Adherence Scale; PSP = public safety personnel. Each model included 12 predictor variables, as described above—this table shows only statistically significant predictors for each model.

**Table 4:** PSP likes and dislikes of ICBT.

Theme	Poster Only,	Poster and Story,	Total,
	*n* (%)	*n* (%)	*n* (%)
**Likes**			
Accessibility			
Time flexible	16 (12)	21 (16)	37 (28)
Convenience	8 (6)	17 (13)	25 (19)
No transportation required	8 (6)	12 (9)	16 (12)
General reference to accessibility	5 (4)	5 (4)	10 (8)
Comfort with technology	4 (3)	6 (5)	10 (8)
No need to schedule or wait for appointment	3 (2)	4 (3)	7 (5)
Enable more people to seek help	5 (4)	1 (1)	6 (5)
Anonymity/privacy	11 (8)	5 (4)	16 (12)
Provides information/techniques/advice	8 (6)	6 (5)	14 (11)
Did not identify any likes	7 (5)	1 (1)	8 (6)
Complements existing treatments	0	4 (3)	4 (3)
Effective	0	3 (2)	3 (2)
Therapist guidance	2 (2)	1 (1)	3 (2)
Brief	0	2 (2)	2 (2)
Low cost/no cost	1 (1)	1 (1)	2 (2)
Breadth of course	1 (1)	0	1 (1)
Interesting	0	1 (1)	1 (1)
Tailored to PSP	1 (1)	0	1 (1)
Team review approach	0	1 (1)	1 (1)
**Dislikes**			
No dislikes identified	23 (17)	23 (17)	46 (35)
Concerns about			
Impersonality or lack of face-to-face contact	20 (15)	21 (16)	41 (31)
Accountability and motivation	5 (4)	9 (7)	14 (11)
Effectiveness	8 (6)	3 (2)	11 (8)
Demands of treatment tasks	1 (1)	4 (3)	5 (4)
Standardized nature of treatment	5 (4)	0	5 (4)
Crisis management	1 (1)	1 (1)	2 (2)
Eligibility	2 (2)	0	2 (2)
Ability to trust e-therapists	0	1 (1)	1 (1)
Confidentiality	1 (1)	0	1 (1)
Triggering negative emotions	0	1 (1)	1 (1)

Note: ICBT = internet-delivered cognitive behaviour therapy; PSP = public safety personnel.

## Appendix A

Educational poster describing ICBT

## Appendix B

Educational story describing a PSP’s experience with ICBT
